# Fuzzy evaluation model for physical education teaching methods in colleges and universities using artificial intelligence

**DOI:** 10.1038/s41598-024-53177-y

**Published:** 2024-02-27

**Authors:** Siyuan Li, Chao Wang, Ying Wang

**Affiliations:** Graduate School, Adamson University, 0900 Manila, Metro Manila Philippines

**Keywords:** Engineering, Mathematics and computing

## Abstract

The evaluation of Physical Education Teaching Methods in Colleges and Universities faces two main challenges: an excess of evaluating elements and a lack of assessment framework. Hence, the research proposes the multi-feature fuzzy evaluation model based on artificial intelligence to streamline the evaluation process and provide an efficient framework for accessing teaching methods. The framework integrates natural/human language using fuzzy instructions considering three evaluation perspectives, including the management stage, instructors, and students and employs the enhanced cuckoo search optimization algorithm. After the teaching expert has determined each parameter's ratings, they are fed into the improved cuckoo search algorithm and solved using an unbiased function to assess the assessment's final result. It incorporates the students' mobility mechanism and movement vector deconstruction designed based on functional criteria. A system for evaluating the quality of instruction has been developed utilizing the proposed model with enhanced cuckoo search optimization. The results indicate that the proposed algorithm has achieved the highest scores across multiple assessment categories, average skill performances of 97.01%, learning progress of 87.36%, physical fitness of 93.49%, participation rate of 95.04%, student satisfaction of 95.49%, and physical education of 96.8% teaching efficiency. The usefulness of the proposed framework in enhancing physical education teaching methods has been demonstrated by comparing the results with traditional methods. It contributes to advancing pedagogical practices in the field.

## Introduction

Artificial Intelligence is a potential response for accelerating and improving the accuracy of PET assessments due to the complexity of the processes and the numerous parameters that must be considered. It is crucial to track and identify motor and cognitive function changes in healthy and unwell individuals, including deaf athletes^1^. AI-based performance analysis is used more frequently to evaluate cognitive and motor skills and motor skills. This AI-based analysis allows for the faster gathering of strategies and associations among data. It has been effective when dealing with ambiguous, imperfect, or data from heterogeneous groups^2^. So, fuzzy models are required to analyze and assess such vague and inadequate data.

Stretching, intermuscular impulses, and inherent muscle characteristics are the processes that sustain posture and prevent it from deviating from the position where the balance of internal and external pressures is preserved^3^. These processes work for each muscle at an appropriate threshold (muscle length, matching joint function). In addition, the aforementioned positional thresholds for particular muscle groups can be changed by spinal and supraspinal centers. This control technique allows us to conclude that a single mechanism is responsible for both positional and kinematic stability that helps the athletes^4^.

Different context-dependent instabilities might result from damage to the postural control system. The posture control processes are computationally modeled to help with the understanding of various neurological controls as well as to help with efficient balance therapy to increase mobility and reduce falls^5^. Although there has been a significant development, fuzzy logic-based techniques for physical activity evaluation are uncommon. Fuzzy logic has been applied to pattern detection in medical signals up to this point^6^, mainly when dealing with language description, ambiguity, and partial data. The beneficial effects of fuzzy logic are anticipated to be most noticeable in injuries, diseases, and deficiencies that require AI's help to be detected early and adequately^7^. Fuzzy logic and agent-based modeling enable the simulation of expert human knowledge, are easy to set up, and offer advanced capabilities (associations and decision-making operations).

The Ministry of Education in China issued recommendations for delivering Physical Education (PE) programs at state colleges and universities in 2002, highlighting the importance of these courses as the primary sources of physical activity for higher-education students. Learners can develop a more robust physique, better their mental and physical wellness, and increase their physical competence through appropriate PE and scientific physical exercise procedures^8^. Therefore, PET assessment is crucial to PE activities, delivering high-quality instruction and fostering healthy growth. Furthermore, to enhance students' psychological and physical development and recognize wellness, PET assessments motivate students to actively engage in physical activity and promptly track and offer observations regarding students' learning impacts, physical appearance, and overall health^9^. Employing advanced computational deep neural network approaches for problem-solving using a Bayesian regularization optimization technique^10^. For addressing nonlinear systems by mathematical modeling and computation studies, AI approaches like artificial neural network (ANN) have been applied^11^ for the accuracy and reliability of the model. The reliability has been calculated using stochastic procedures approved through statistical populations identified for training the model in addition to the need for an optimization algorithm^12^. The learning parameters were employed and fine-tuned for complexity analysis initiated with the population and local search policies^13^.

An objective, efficient, and precise technique to assess the efficacy of Physical Education Teaching Methods in Colleges and Universities (PETCU) may be found by creating a fuzzy assessment model for such methods using AI. The standard of PE can be improved and encourage students to lead healthier lives by utilizing AI technologies.

The proposed MFEM based on AI addressed challenges like intuitive communication, accommodating imprecise information, and enhancing the efficiency of teaching assessment in PETCU. The model, guided by expert input, considers student dynamics and employs a streamlined system architecture. Resulting in consistently high scores across various assessment categories, the Multi-feature Fuzzy Evaluation Model based on Artificial Intelligence (MFEM-AI) outperforms traditional methods, providing a comprehensive and effective framework for evaluating and improving physical education teaching methods.

The research addresses challenges in evaluating PETCU, focusing on an overload of evaluation elements and a lack of a clear assessment framework. The proposed solution is the MFEM-AI, aiming to streamline the process and provide an efficient framework. The model integrates natural language, fuzzy control instructions, and an Enhanced Cuckoo Search Optimization (ECSO) algorithm to enhance the evaluation of teaching methods in physical education.

The novelty of the research lies in its integration of AI, fuzzy logic, and ECSO for assessing the quality of physical education teaching. It enhances the precision of the proposed model's evaluation. The model proposes a comprehensive evaluation framework from educational institutions, instructors, and student perspectives. The models' potential capability for diverse physical education makes it innovative. Promising performance comparison across various assessment dimensions with benchmark schemes offers a distinct approach.

The following are the main contributions of this paper:

1. To propose a Multi-feature Fuzzy Evaluation Model based on Artificial Intelligence (MFEM-AI) to effectively implement PET methods by combining natural/human language with Machine Learning (ML) using fuzzy control instructions.
2. To build an AI-based teaching evaluation index system for PETCU with five fundamental features.
3. Using the improved cuckoo search optimization technique, the system architecture has been created using the analysis of the functional criteria to record the student's mobility mechanism and movement vector deconstruction.
4. The proposed MFEM-AI model with enhanced cuckoo search optimization has been used to construct a teaching quality evaluation framework for PETCU.
5. Comparing the results of MFEM-AI to those of traditional PET methodologies demonstrates the usefulness of the suggested framework.

The remainder of the article has been structured as follows: second section describes related research on PETCU evaluation using fuzzy systems and AI. Third section provides a Multi-feature Fuzzy Evaluation Model based on Artificial Intelligence (MFEM-AI). Results and discussion have been given in fourth section. Finally, the conclusion, limitations, and scope for further study have been demonstrated in fifth section.

## Related works on PETCU evaluation using the fuzzy system and AI

An Internet of Things (IoT) evaluation technique for computer-based teaching quality assessment was suggested by Huang^14^. The proposed approach uses IoT technology to gather and evaluate current student conduct and achievement information, resulting in a more precise and effective evaluation of teaching quality. The findings demonstrated that the suggested strategy could increase the efficacy and accuracy of evaluating the quality of instruction. However, the expense of installing IoT technology may be a barrier, and the approach may need an advanced degree of technical competence. The Technique for Order Preference by Similarity to Ideal Solution (TOPSIS) technique, published by authors in^15^, is an intuitive, fuzzy approach for evaluating the quality of PET. The suggested approach considers objective and personal evaluation factors to give a more thorough assessment of teaching performance. The findings demonstrated that the proposed technique might accurately assess the standard of PET. However, the procedure could need a lot of information input, which could take a while.

Gao et al.^16^ briefly described essential technologies in AI and big data-driven e-learning and e-education. Then, the essay highlights how customized and flexible educational experiences might revolutionize education by utilizing AI and big data. Next, the paper emphasizes how crucial it is to address the moral, confidentiality, and security issues raised using big data and AI in education. Finally, the article summarizes the most recent developments in AI and big data-driven education.

Based on the standpoint of the theory of self-determination, Chiu and Chai^17^ suggested an ecologically sound instructional framework for AI in PET. The proposed paradigm highlights the significance of students' independence, competence, and resemblance to promote long-term inspiration and participation in AI learning. The outcomes demonstrated that the suggested framework could raise students' excitement and drive to learn PE. Nevertheless, the framework would need further validation and improvement in light of various educational environments and student groups.

Yuan et al.^18^ suggested a movement sensor-based basketball technique and a neural network-based system for evaluating physical fitness. Motion sensors are used in the system recommended to gather and assess information regarding basketball skills and physical condition, resulting in a more precise and impartial performance assessment. The findings demonstrated that the suggested approach might accurately assess basketball abilities and physical condition. However, the precision and caliber of the motion detector's data may impact the system's performance.

Kong^19^ suggested using AI technology in teaching current art. The suggested method uses AI algorithms to produce individualized art classes and feedback depending on the traits and requirements of specific students. The outcomes demonstrated that the recommended strategy could increase pupil innovation and involvement in art education. However, the technique could need more validation and improvement depending on various art education settings and student groups. Nevertheless, the suggested method illustrates how AI technology in art education might improve students' educational outcomes.

A mobile edge computing-based technique for evaluating the quality of combined online and offline PET was proposed by Bao and Yu^20^. The suggested approach takes advantage of the real-time data collection and processing capabilities of mobile edge computing to offer a more effective and precise assessment of teaching quality. The findings demonstrated that the suggested strategy could increase the efficacy and accuracy of evaluating the quality of PET. However, the system could need a high degree of technical know-how, and the expense of implementing mobile edge computing technologies might be a barrier.

Liu^21^ suggested an assessment of PET based on AI and machine vision. The suggested approach uses machine vision technology to examine how students move and behave in PE sessions, giving a more precise and impartial assessment of the quality of instruction. The findings demonstrated that the suggested technique might accurately assess the standard of PET. However, the strategy could need advanced technical experience, and the expense of implementing machine vision technology might be a barrier. AI technology may be used to increase evaluation precision and effectiveness in the suggested technique.

Under the guidelines of inclusive education, Demchenko et al.^22^ suggested a training curriculum for prospective PET. The proposed program combines academic knowledge, hands-on experience, and multicultural teaching techniques to provide aspiring teachers with the skills they need to deal with various student demographics. The outcomes demonstrated that the suggested approach might successfully train aspiring teachers for occupations in inclusive settings. The success of the curriculum, however, may differ based on the particular situation and features of the student body.

Huang et al.^23^ elaborated physical education training program impact of a student's fitness related to injury risk in the basketball team of college was analyzed using linear discriminant analysis (LDA) as a main feature extraction technique. The prediction of fitness assessment of students' attributes on agility and speed was based on a cost-effective neural network approach with precision, recall, f1-score, and area under the curve was predicted as 63.6%, 87%, 79.8%, and 85.95. Even though the study supported fatigue management and injury prevention, it still lacks many variables for physical assessment.

Zong^24^ proposed PE teaching quality evaluation using a convolutional neural network (CNN) with multi-dimensional data from teaching attributes like activities and psychological analysis through the Internet of Things (IoT). The evaluation criteria include emotional level, self-challenge, student training ability, and practical approach. The result iteration after counts produced the degree of satisfaction of 89% evaluation before and after evaluation with the error value of 0.176. The efficiency of the proposed approach depends on the curriculum reform in physical education, which is not practically possible in real-time.

Jiao et al.^25^ defined a new method for PE course score prediction using a principal component analysis (PCA) based deep neural network with a factorization technique that impacts the student grade level. For evaluating the PE teaching quality and students' learning efficiency produced the recall as 95%, precision as 86.8%, area under the curve as 82.9%, and accuracy as 86.6%. The analyzed number of attributes for performance prediction of students based on teaching quality was very small.

Sun and Ma^26^ applied the Naïve Bayes (NB) classification algorithm for improving the physical education teaching quality to analyze the physical fitness among college students. The result proved that 81.02% of the physical classifier rate was achieved using the slicing technique on attributes capacity, age, grade, power, strength, and body mass index as excellent, good, pass, and fail categories. The accuracy achieved was 82%, and the training scenario was different for each student.

Xue^27^ elaborated on the teaching quality of PE using an enhanced random forest (RF) classifier adopted in an energy-efficient based routing technique in a scalable manner in a wireless environment. The analyzed attributes of 3000+ students produce increased strength, speed, and quality with an involved student engagement level of up to 94.6%, quality of PE teaching was 90%, precision of 96.8%, recall of 98.3%, and 98.1% f1-score. The proposed strategy has opted only for online teaching and is impractical for getting improvement offline.

Chen et al.^28^ aimed to improve the teaching assessment of physical education classes with the fuzzy neural network (FNN) in a multi-index evaluation system for college physical education with an analytical hierarchical technique. The evaluation of teaching staff includes teaching content, method, attitude, and influence. The output of this study indicates the quality of college physical education employed by the students termed as good, average, and bad. The evaluated study demonstrated accuracy as 95%, specificity as 94%, sensitivity as 92%, and F1-score as 93%. The study has a lack of consideration towards influencing factors of the teaching management of physical education classes.

Zhu^29^ proposed the teaching system of college physical education learning using an ANN variant called backpropagation (BP) neural network. The 10 influencing factors of the college teaching system were optimized using a genetic algorithm (GA) with the variant of curriculum, skills, and learning style of education courses. The demonstrated results showed an average error detection of about 1.23, and the average error rate of 1.85% leads to sports innovation using advanced AI techniques. The reported study should consider the change of assessment towards teaching among students.

Guo^30^ promoted college physical education online courses using deep learning-based 3D reconstruction techniques for assessment tasks and training exercises. Using foreground and background features, high and low-resolution multi-scale features were analyzed using a deep clustering layer (DCL). The study demonstrated the execution of several iterations reached 120 epochs with enhanced accuracy and minimal loss rate. The challenge of uncertainty in the reconstruction of human back under single-view condition on the sports lessons.

Li^31^ studied the feasibility of physical education teaching among college students using cluster analysis of the K-medoids (C-K) algorithm along with the AI model namely the semi-supervised radial basis function neural network (RBF-N2). The performance of teaching comparison in standing long jump and 50 m was analyzed with statistically significant results with p values < 0.05 with no significant difference related to physical fitness education.

In conclusion, assessment models based on fuzzy logic and AI have demonstrated favorable outcomes in assessing the effectiveness of PET techniques. The suggested algorithmic approaches offer a more thorough and precise assessment of instruction quality, which can help increase the efficiency of PETCU. Additional research is necessary to verify and improve the suggested models and methods in various scenarios and student demographics.

## Proposed multi-feature fuzzy evaluation model based on artificial intelligence

The section proposes the creation of a multi-feature fuzzy evaluation model that utilizes artificial intelligence to facilitate the implementation of PET techniques. The multi-features of physical education are students' motor abilities like strength, endurance, speed, flexibility, coordination, agility, and balance. This is achieved by integrating natural language with machine learning through fuzzy control instructions. Finally, this section outlines developing a teaching evaluation index system for PETCU that utilizes an AI-based approach. The system is constructed using an enhanced cuckoo search optimization technique, a student's mobility mechanism, and movement vector deconstruction to establish the system architecture. The outcome is a framework for evaluating teaching quality at PETCU. The evaluation framework integrates a three-layered architecture with the ECSO algorithm to assess physical education teaching. It considers multiple features, uses fuzzy control, and evaluates from management, instructor, and student perspectives. The ECSO algorithm optimizes AI model parameters, contributing to intelligent assessments. The system ensures comprehensive evaluation, including data preprocessing, multi-feature assessment, and a structured ECSO workflow. The proposed MFEM-AI framework enhances the precision and consistency of instructional evaluations in physical education.

### System model for accessing the quality of physical education teaching at PETCU

The basic system model for the proposed MFEM-AI for accessing the physical education quality at PETCU involves several processes. The MFEM-AI framework employs fuzzy logic, a rule-based system, and membership functions to handle imprecise linguistic variables in teaching evaluations. Fuzzy control instructions guide the evaluation process, allowing for adaptive decision-making and flexibility in interpreting natural language patterns. The approach enhances communication by enabling the system to understand and respond to the nuanced language used by teaching experts, making the evaluation process more intuitive and user-friendly.

#### Data acquisition and preprocessing

The system collects data related to physical education quality information on students' motor abilities like strength, endurance, speed analysis, flexibility, coordination, agility, and balance. The data preprocessing technique involves data cleaning and normalization steps in subsequent steps.

#### Multi-feature assessment

The system assesses multiple features related to physical education, with a focus on student's motor abilities. These identified features serve as the foundation for the evaluation process.

A fair evaluation of physical education quality necessitates establishing a systematic structure for evaluating indicators. The conventional method for assessing the quality of teaching includes two main categories, namely comprehensive teaching subject matter and comprehensive achievement in school, to establish the instructing index and assessment index. The fundamental aspects of the indications utilized in traditional assessment systems are their feasibility, representativeness, and autonomy. These factors serve as the foundational elements for the types above of traditional assessment system indications. The proposed MFEM-AI is executed through a process that involves integrating natural language and fuzzy logic. Teaching experts assign ratings to parameters, and the improved cuckoo search algorithm optimizes these ratings. The model's efficiency is ensured through a carefully designed system architecture. MFEM-AI consistently outperforms traditional methods in evaluating physical education teaching, scoring high across various assessment categories, including skill performance, learning progress, physical fitness, participation rate, student satisfaction, and overall teaching efficiency.

### System architecture

The system architecture is designed to manage data flow in three types of layers efficiently and executes the model with feedback. The architectural components include the data management layer, called the feature layer, the indication layer, and the AI evaluation layer, called the target layer. The ECSO is the core of the architecture for evaluating the optimization procedures for fine-tuning the fuzzy parameters for optimal solution of physical education quality.

The creation of the system architecture considers functional criteria related to evaluating physical education methods, including data acquisition and preprocessing, multi-feature assessment focusing on student's motor abilities, a systematic structure for evaluating indicators, and a three-layered architecture comprising a data management layer (feature layer), an indication layer, and an AI evaluation layer (target layer). The architecture integrates the ECSO algorithm for fine-tuning fuzzy parameters and optimizing the assessment model. The system aims to enhance reliability and efficiency in evaluating teaching quality at colleges and universities.

**Figure 1:**
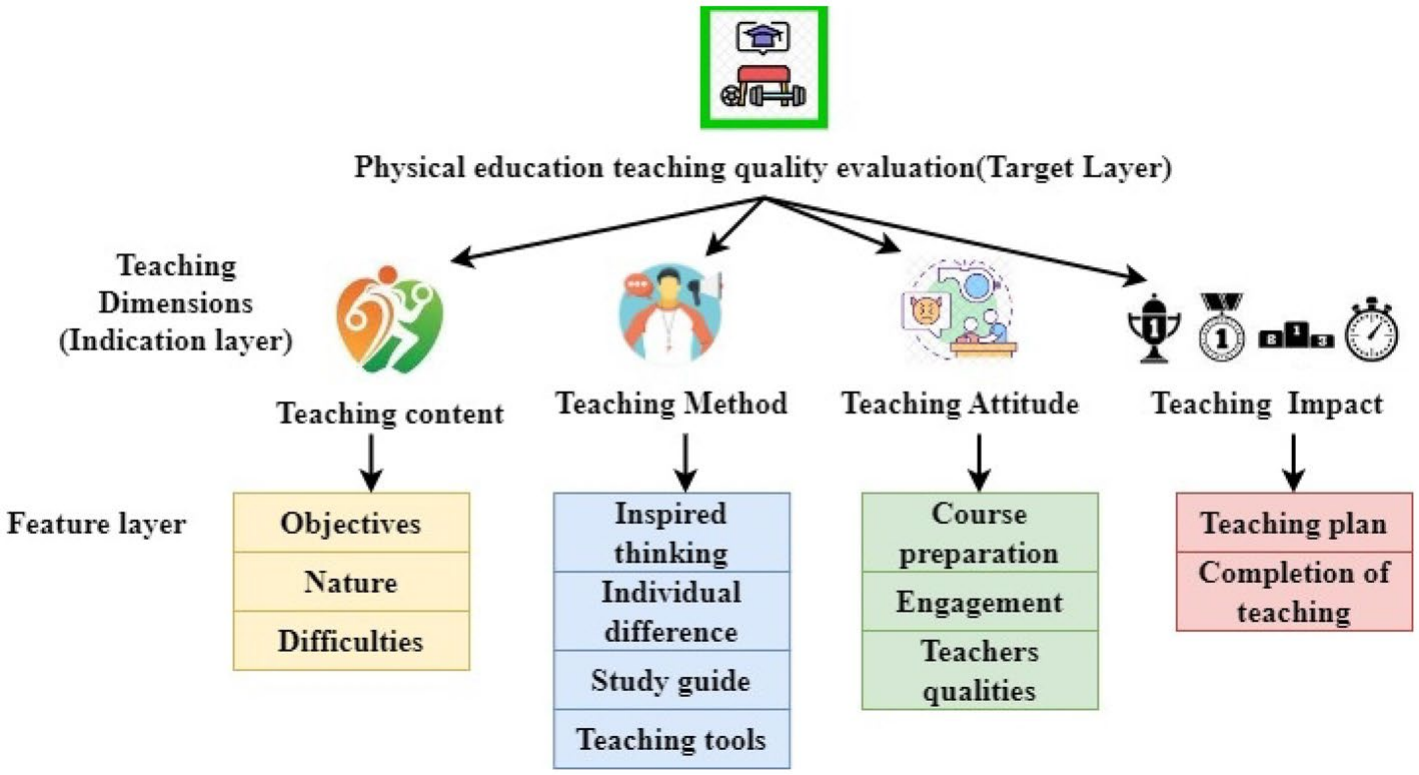
The layered architecture of the proposed MFEM-AI.

This study constructs a multi-index evaluation system for PE in colleges using the analytic hierarchy process (AHP) methodology. The evaluation method depends on four dimensions: teaching method, teaching content, teaching mentality, and teaching impact. The layered architecture is displayed in Fig. 1.

**Figure 2:**
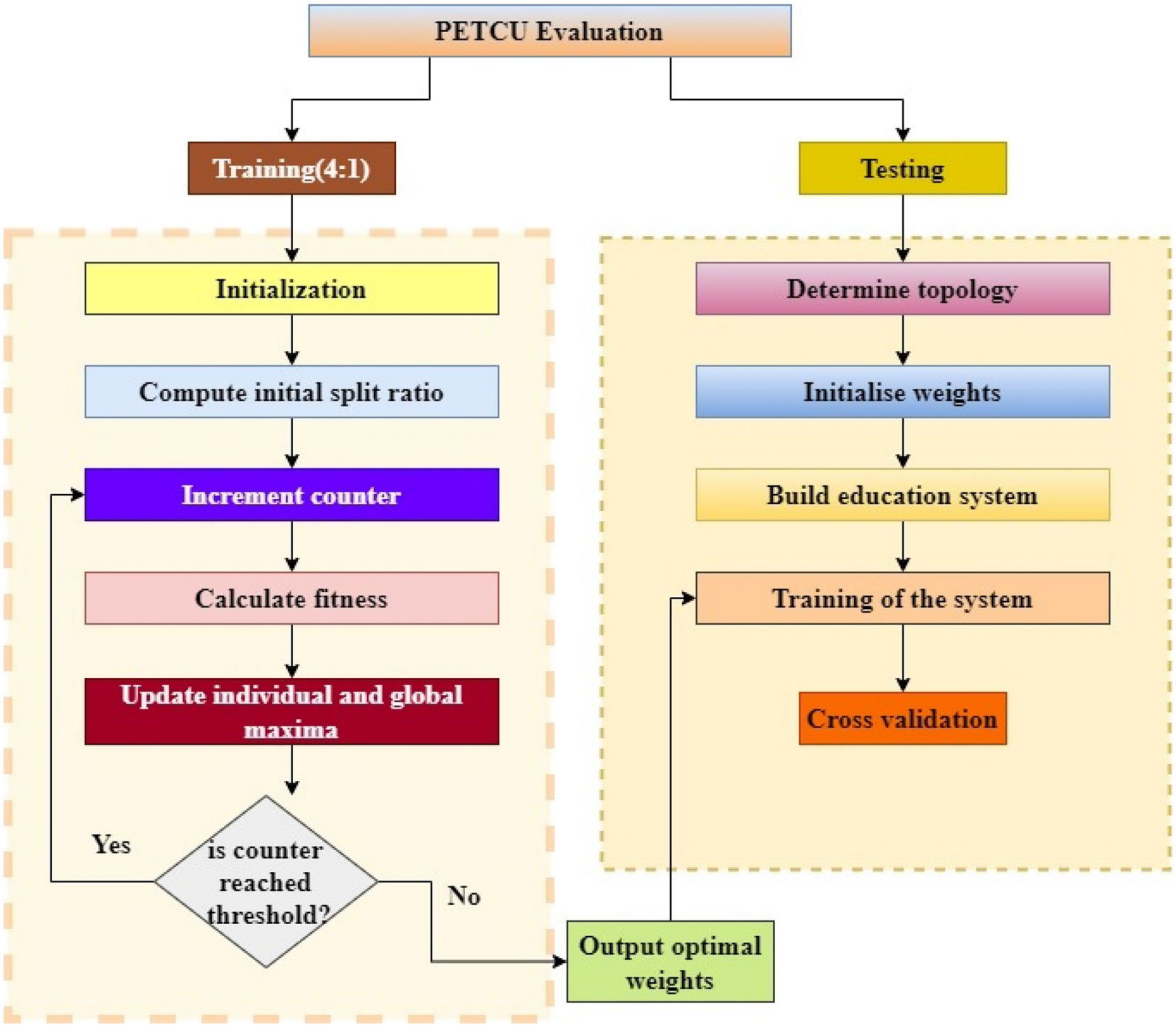
Cuckoo search optimization workflow.

The procedure for implementing the Enhanced Cuckoo Search Optimization (ECSO) based quality of instruction evaluation approach for sports departments in universities is illustrated in Fig. 2. Initially, the database sourced from the evaluation system for physical education (PE) teaching quality in tertiary institutions is partitioned into two distinct sets, namely the sets for training and testing, with a ratio of 4:1. The present study employs a joint nerve-tailored Enhanced Cuckoo Search Optimization (ECSO) approach, as proposed in the literature, to construct a model for evaluating physical education instruction in higher education institutions. The optimal setting of weights and limits is achieved through joint nerve optimization. To conduct testing, the ECSO model should be utilized for insertion. The subsequent text presents a comprehensive, sequential analysis of the execution procedure.

1. Stage 1: The data about the PE ratings of colleges and universities ought to be scrutinized academically. Specifically, it should be partitioned into learning and training sets, with a ratio of 4:1, and subsequently normalized using Eq. (1).
  1 $${O}_{x+1}={T}_{h}+\frac{o-{o}_{min}}{{o}_{max}-{o}_{min}}+{T}_{f}-{T}_{h}$$
  The available datasets consist of ${O}_{x+1}$, which denotes the normalized information and ${o}_{min}$ and ${o}_{max}$, which respectively indicate the minimum and highest values visible in the ${O}_{x+1}$ dataset. The dataset that has been normalized exhibits a range of values between the most negligible values (${T}_{f}$) and the highest value (${T}_{h}$), with ${T}_{h}$ being assigned a value of -1 and ${T}_{f}$ being assigned a value of 1.
2. **Stage 2:** Initiate the primary variables of the joint nerve approach, including h and f, ${S}_{max}$, P and Q. The first step is to set up the structure of the fuzzy system, followed by the initialization of the weighting and sensitivity.
3. Stage ****3: Eq. (2) is utilized to initiate the joint nerve method group, followed by applying the Fuzzy model to obtain weights and limits. This ensures that all individuals in each population are initialized at a uniform starting point.
  2 $${P}_{x}={T}_{f(x)}+rand\left(\mathrm{0,1}\right)\left({g}_{f(x)}-{T}_{f(x)}\right)$$
  The initial value of a person is denoted as ${P}_{x}$, while the upper and reduced search boundaries of the x person are represented by ${T}_{f(x)}$ and ${g}_{f(x)}$, accordingly.
4. **Stage 4:** Compute ${O}_{1}$ and ${O}_{2}$ for the joint section coefficients utilizing Eqs. (3) and (4) in the following manner:
  3 $${O}_{1}=c\left(1-\propto \right)+f\propto $$
  4 $${O}_{2}=c\propto +f\left(1-\propto \right)$$
  The scaling factor is denoted $\propto$, the coefficients are denoted $c$, and the function is denoted $f.$
5. **Stage 5:** Compute the optimal fitness value, denoted as ACC, for each population member using Eq. (5).
  5 $${\text{max}}\left(y,v\right)A=\frac{\sum_{x=0}^{N}A(x)}{I}$$
  $A$ represents the mean value of K-fold cross-validation reliability. In contrast,$A(x)$ denotes the mean accuracy values obtained from K-fold calculations.
6. **Stage 6:** It is recommended that individual positions be modified to reflect current circumstances accurately. The unique position is denoted in Eq. (6).
  6 $${S}^{u+1}\left(x\right)={S}^{u}\left(x\right)\left|{\text{sin}}\left({y}_{1}|-{y}_{2}{\text{sin}}\left({y}_{1}\right)\right)\right|\left|{o}_{1}{T}^{u}\left(x\right)-{o}_{2}{S}^{u}\left(x\right)\right|$$
  The individual output is denoted ${y}_{1} and {y}_{2}$. The fitness function is denoted ${T}^{u}\left(x\right)$, and the score is denoted ${S}^{u}\left(x\right)$. The final updated score is denoted ${S}^{u+1}\left(x\right)$.
7. **Stage 7:** Compute the fitness value ${A}_{x+1}$ of the calculators and juxtapose it with the optimal fitness value ${A}_{x+1}$ of the preceding generation for the populace constituents whose locations have been altered. To obtain the most up-to-date fitness value, it is imperative to update the optimal fitness value to the present repetition if ${A}_{x+1}$ surpasses ${A}_{best}$. It is recommended that ${A}_{best}$ be modified to accurately reflect the user's present location while maintaining its static nature in all other aspects.
8. **Stage 8:** To verify whether a method has satisfied its termination circumstance, it is necessary to examine the present cycle count t and compare it against the maximum allowable value ${R}_{max}$. If the condition is met, it is recommended to cease all ongoing operations and produce an optimal fitness value and an ideal location.
9. **Stage 9:** Conducting a quality evaluation of physical education pedagogy in higher education institutions, utilizing the results obtained from stage 8's results.

### College PE teaching evaluation index

To enhance the reliability of evaluation outcomes about the quality of physical education instruction in higher education institutions, it is imperative to undertake a comprehensive assessment and analysis of the instructional standards of PETCU. Therefore, this paper examines the PETCU from three evaluation perspectives: the management stage, instructors, and pupils. It is worth noting that each viewpoint emphasizes different aspects of the assessment process.

#### ${E}_{1}$, from a managerial perspective, the assessment method for physical education instructional excellence in college can be categorized into sub-levels

The evaluation system denoted as ${E}_{1},$ primarily examines the influence of the fundamental physical education amenities of the college on the standard of instruction as viewed through the lens of the administration. This study undertakes an extensive assessment encompassing various aspects, including the professional competencies of physical education (PE) instructors, denoted as ${E}_{11}$, the comprehensiveness of college-level PE courses, designated as ${E}_{12}$, the capacity for reform in college-level PE instruction indicated as ${E}_{13}$, the adequacy of software and hardware establishments and expenditures on PE instruction characterized as ${E}_{14}$, and the ability to integrate PE instruction within the industry-college-research cooperation framework denoted as ${E}_{15}$.

#### ${{\text{E}}}_{2}$, the present study examines the sub-level assessment structure of college physical education teaching quality from the instructors' perspective

The ${E}_{2}$ assessment framework primarily examines the effects of implementing the PE instructional process on the overall quality of PE, as perceived by educational professionals. This study undertakes a comprehensive assessment encompassing several dimensions, including the originality and depth of the PE curriculum ${E}_{21}$, the logical and empirical basis of PE lesson plans ${E}_{22}$, the efficacy and flexibility of PE instructional techniques ${E}_{23}$, the variety and cognitive complexity of PE pedagogies ${E}_{24}$, the fulfillment of PE teaching objectives ${E}_{25}$, and the instructional quality and impact of PE classes ${E}_{26}$.

#### ${E}_{3}$, the present study focuses on assessing college physical education teaching quality through a sub-level evaluation structure from the pupils' perspective

The ${E}_{3}$ assessment system primarily examines the outcomes of physical education instruction in higher education institutions, focusing on the student context. This study undertakes an in-depth evaluation of various factors, including the success rate of pupils in the ${E}_{31}$ broad sports quality test, the unprecedented rate of pupils in the ${E}_{32} general$ sports quality examination, the creative capacity of pupils as measured by ${E}_{33}$, the independent learning ability of pupils as measured by ${E}_{34}$, the community serviceability of pupils as measured by ${E}_{35}$, and the rewards received by students in competitive sports as measured by ${E}_{36}$.

### Enhanced cuckoo search optimization

A group of elements comprising n units is placed in a D-dimensional space to determine the most efficient solution. The i-th egg's location is denoted as A, while its velocity is represented by B. Simultaneously, the trajectory of the egg's motion ought to take into account both the egg's historical optimal position (${P}_{best}$) and the ECSO optimal location for the grouping (${P}_{best}$). The ECSO optimizing issue can be solved by determining the feasible fix of the egg location.

The variable x is introduced into the given issue, and the outcomes are contrasted to assess the optimization level achieved by the viable solution. ECSO compatibility is a term commonly employed to describe the advantages and disadvantages of feasible solutions. In instances where the objective work necessitates minimal value, the ECSO optimal solution would be the one that is possible with the lowest egg appropriateness.

Eggs utilize an iterative calculation to modify their velocity and location based on all three tenets of motion. This ECSO process continues until the elements reach the optimum state under the circumstances at hand. The updated optimization result is denoted in Eq. (7):

7 $${s}_{x}^{t+1}={w}_{x}^{t}+{k}_{1}{q}_{1}\left({p}_{x}^{t}-{i}_{x}^{t}\right)+{k}_{2}{q}_{2}\left({p}_{y}^{t}-{i}_{y}^{t}\right)$$

The variables, $y, w, t, {k}_{1}$, ${k}_{2}$, ${q}_{1}$, and ${q}_{2}$ are utilized in ECSO. Specifically, $x$ is a discrete parameter that denotes the number of elements in the swarm, while $y$ reflects its spatial dimension. The momentum weight is represented by $w, and t$ is an integer that signifies the number of repetitions. Additionally,${k}_{1}$ and ${k}_{2}$ are learning variables, and ${q}_{1}$ and ${q}_{2}$ are arbitrary numbers that fall within the range of [0, 1].

The composition of ECSO egg velocity is primarily comprised of three distinct components. The initial component comprises the legacy velocity ${S}_{x}$ from the preceding repetition, a historical account of the egg's motion. The following element pertains to the ECSO egg's ideal location ${V}_{p}$, which serves as a point reference point for the egg's self-experience and guides the optimization procedure. The final component pertains to the group's ideal location S. The process of sharing ECSO information embodies the benefit of the population, which significantly aids individual eggs in locating the most effective approach.

ECSO stands out among other population methods and smart optimizing methods due to its notable advantage of requiring fewer variables to be selected while maintaining high result reliability. The rate of acceleration is comparatively higher due to the information exchange system. The computerized control system exhibits a higher level of ECSO responsiveness. The employed ECSO algorithm optimizes parameters for assessing students' physical abilities in physical education. It fine-tunes the factors related to mobility mechanisms, including endurance, speed, flexibility, coordination, agility, and balance, to analyze how well students perform in terms of these physical attributes. Deconstructing the movement vector involves analyzing how each of the historical information, individual solution preferences, and overall population trends contributes to the changes in solution positions, providing insights for optimizing the evaluation process.

The present study outlines the key variables used in the ECSO method, which include the egg size denoted by n, the weight of the inertia represented by w, the learning variables ${k}_{1}$, and ${k}_{2}$, the highest speed ${S}_{max}$, the slowest speed ${S}_{min}$, the iterative number 't', and the egg suitability indicated by $\sigma$. Equation (8) is used for reducing inertia.

8 $$w\left(x+1\right)={w}_{init}-\frac{{w}_{init}-{w}_{last}}{{t}_{max}}t$$

The variables under consideration include t, which denotes the present iteration count, ${t}_{max}$, which represents the upper limit of the repetition count, ${w}_{init}$, denoting the starting inertia weight; and ${w}_{last}$, representing the ultimate inertia weight for the nest.

#### Learning factors ${{\varvec{k}}}_{1},{{\varvec{k}}}_{2}$

The learning variables ${k}_{1}$ and ${k}_{2}$ is commonly called accelerated unchanged speed in academic literature. These factors signify the relative significance of the egg's individual experience and collective participation in the investigation of motion.

A value of ${k}_{1}$ equal to zero indicates that the egg's personal experience does not contribute to the optimization process of identifying the best possible outcome. Currently, the egg swarm algorithm solely incorporates the notion of a collective, resulting in a quicker convergence rate than the conventional ECSO method. However, its efficacy in addressing intricate problems remains to be determined. Due to the lack of empirical evidence supporting specific eggs, there is a greater likelihood of observing a concentration of all fragments around one or a few extremes. The answer gathered can be a local extreme rather than a global optimum.

In the scenario where ${k}_{2}$ equals zero, there exists a lack of communication regarding group data, and the collective experience of the entire population does not influence the egg's motion. Currently, the benefits of the population method have yet to be manifested, as all eggs are undergoing individual action, resulting in a sluggish running pace for reaching the nest. Identifying the best answer for the goal function poses a challenging task.

#### Maximum speed ${{\varvec{S}}}_{{\varvec{m}}{\varvec{a}}{\varvec{x}}}$

The variable ${S}_{max}$ denotes the highest attainable velocity of an egg within a D-dimensional space during a single iteration, corresponding to the egg's most significant possible displacement. If the value of ${S}_{max}$ is excessively high, the ECSO egg should fail to converge on the most effective region. Conversely, if the value of ${S}_{max}$ is excessively low, not only will the solution's acceleration be impeded, but there is also a possibility of solely identifying the local extrema rather than the optimal global solution. In the D-dimensional area, it is common practice to establish the ${S}_{max}$ value as equivalent to one-fifth of the disparity between the top and bottom limits. The mathematical equation is represented in Eq. (9).

9 $${S}_{max}=\frac{{s}_{max}-{s}_{min}}{n}$$

The minimum and maximum solutions are denoted ${s}_{min}$ and ${s}_{max}$. The total number of solutions is denoted $n$.

#### Minimum speed ${{\varvec{S}}}_{{\varvec{m}}{\varvec{i}}{\varvec{n}}}$

The lowest velocity refers to the shortest distance each egg travels during its movement, guaranteeing the operational speed. Typically, the value assigned to ${S}_{min}$ is the negation of the highest rate, denoted as ${S}_{max}$, i.e., ${S}_{min}=-{S}_{max}$.

#### The number of iterations t

The method of ECSO persistently endeavors to attain the most favorable solution using the iterative modification of eggs. It conventionally concludes the process by establishing the number of repetitions.

#### Optimization with ECSO

The ECSO algorithm is a metaheuristic optimization technique used to optimize AI model parameters. This involves adjusting parameters like weights, biases, or hyperparameters to improve the model's performance. The system uses the ECSO technique to optimize and fine-tune the parameters of the AI models. This optimization step is crucial for achieving accurate assessments. Equation (7) shows how ECSO updates egg positions based on velocity and historical information.

#### ECSO suitability ${\varvec{\sigma}}$

The practicality of an answer can be succinctly expressed as the magnitude of the discrepancy between the real optimal solution and the idealized optimal solution. The attainment of the optimizing objective is evaluated through the computation of the suitability metric. The standardized variables for calculating nest variables using Eq. (10).

10 $$Q=\frac{2}{\left|2-k-\sqrt{{k}^{2}-4k}\right|}$$

The egg velocity is denoted $as k$. The development and analysis of an assessment system for performance for physical education instruction. The parameters of the ECSO algorithm involve population size ${P}_{x}$ that gives the number of nests the maximum number of iterations, followed by the learning variables ${k}_{1}$ and ${k}_{2}$ to determine the relative importance of individual experience and group participation, the maximum attainable velocity of an egg ${S}_{max}$. Likewise, the shortest distance each egg travels during its movement ${S}_{min}$, the no. of iterations in the optimization process is given as $t$, and the initial and last inertia weight is represented as ${w}_{init}-{w}_{last}$, those matches with the termination criteria are analyzed for ECSO suitability factor $\sigma$.

This study has constructed a physical education instructing assessment system utilizing the artificial intelligence fuzzy method with the methods and automobiles. The ECSO algorithm is shown in Table 1.

**Table 1:** ECSO algorithm.

Step 1	Initialize parameters
Step 2	Define the fitness function
Step 3	Evaluate the fuzzy model with teaching method x
Step 4	Return fitness score based on evaluation criteria
Step 5	Initialize eggs and nests
Step 6	Initialize personal bests and global best
Step 7	Start iterations
	Update velocity
	Update position
	Update personal best
	Update levy flight
Step 8	Return the best teaching method found

Before instruction, training students on properly utilizing said tools is imperative. Using instruction, learners can acquire comprehension and proficiency in the procedures and stages of the complete process of online course education, which encompasses downloading and setting up of the system, authorization, and authentication, selection of classes, viewing of videos, perusal, and retrieval of textual substances, utilization of message boards for debate, and fulfillment of online tasks and tests. In addition, these training sessions facilitate pupils adapting to teaching and establishing a solid groundwork for the seamless progression of their subsequent instructing endeavors.

### ECSO algorithm workflow for optimizing the assessment model

The system utilizes the ECSO algorithm to optimize the assessment models. This includes setting parameters, evaluating fitness functions, and updating the next positions for optimal evaluation. The algorithm's initialization process starts with the parameters like population size, maximum iterations, and learning variables. The velocity and position update is performed for each egg in the nest. It optimizes these parameters to approach the best solution. The algorithm also includes a termination criterion, such as reaching a maximum number of iterations or achieving a satisfactory solution. The best solutions found during the optimization process are recorded for further assessment of physical education.

The methodological analysis presented above indicates that using the ECSO model enhances the CS algorithm. The optimal input weights and limits are determined by manipulating the hidden layer node count in machine learning (ML). These are then contrasted with the most optimal location produced by the Cuckoo Search (CS) algorithm. This process enhances the precision and consistency of instructional evaluations while reducing the duration of training. The procedural steps for implementing the strategy above are as follows: initially, the Intelligent Instruction Effect Assessment Index is formulated by incorporating five key dimensions, namely fundamental quality, teaching demeanor, pedagogical approach, instructional proficiency, and teaching outcome. Subsequently, a professional evaluation determines each assessment index's score and ultimate rating. Later, the evaluation indices' scores are utilized as the input for MFEM-AI, with the resulting output being the top score. A model for evaluating the teaching effectiveness of MFEM-AI has been developed, demonstrating a high level of intelligence. Finally, the expert assessment technique is utilized to obtain the scores for every assessment index and the complete scores of the innovative teaching impact assessment. The evaluation indices have been assigned scores of 1, 0.7, 0.5, 0.3, and 0.1, which match the levels of outstanding, acceptable, medium, poor, and poor, accordingly. The structure of AI used in this research is not focused on the typical neuron structures; rather, it is used to access physical education teaching quality through fuzzy control instructions with the ECSO algorithm.

#### Initialization

The assessment data about the effectiveness of philosophical teaching will be read based on the index structure. Subsequently, the data is to be segregated into a set for training and a test set, followed by normalization. The parameters such as population size, maximum iterations, and learning variables are set. The range of egg velocities is defined with the initialized number of iterations.

#### Iterative optimization

The present study outlines the configuration of variables for the ECSO algorithm, which includes the number of structures denoted by N, the maximum amount of repetitions represented by M, and the likelihood of outside birds' eggs being detected by the nest support, characterized by ${P}_{x}$. Compute the values of all objective functions about the nests. The algorithm updates the velocity and position of each egg in the nest. Velocity is updated based on historical information, an egg's ideal location, and the group's ideal location. The fitness of each egg is calculated using a fitness function that quantifies the quality of evaluation of PE teaching.

#### Optimization process

The procedure involves updating the nest's location, computing the unbiased function appreciation of the nest post-update, and subsequently comparing it with the neutral operate value before the revision. The nest with the prime objective operate value is then selected as the present place. The algorithm iteratively updates the egg positions, optimizing them to approach the best solution. A termination criterion is checked, such as reaching a maximum number of iterations or achieving a satisfactory solution.

#### Algorithm termination criteria

Produce a random variable, denoted as r, such that r belongs to the open interval (0,1), to conduct uniform analysis. It is assumed that r is more significant than ${P}_{x}$. Revise the current nest position, evaluate the objective function parameters for all nests, and retain the nest position that yields the most effective accurate function value. After achieving the function criteria, the algorithm stops, and the optimal solution and its location are recorded. The stopping criteria of the proposed ECSO network stop when the following conditions are met that is a random variable $r$ is generated in the open interval (0,1), and it is compared to a threshold ${P}_{x}$. If $r>{P}_{x}$, the algorithm revises the current position of the nest, and the algorithm stops after reaching the function criteria where an optimal solution with its location is recorded.

#### Revert to fitness evaluation

Ascertain the termination of the method. Upon satisfaction of the termination circumstance, the optimal solution from the historical data is duly documented. Alternatively, revert to Step 3. The fitness value for each egg in the population is determined with conditions and checks for teaching quality.

#### Evaluation result

The optimal positioning of the bird's nest is contingent upon the optimal values of the starting input weight ${w}_{x}$ and concealed layer bias ${b}_{x}$ in the AI system. These optimized parameters are crucial for accurate assessments of PE quality. Similarly, the optimal values of the initial participation weight ${b}_{x}$ and concealed layer bias ${w}_{x}$ are utilized in the ECSO model to assess the efficacy of innovative classes.

The ML evaluation model, coupled with the ECSO, addresses parameters from PET experts by integrating fuzzy instructions. The ECSO optimizes these parameters through a comprehensive process, providing a systematic and unbiased evaluation of physical education teaching methods, ultimately leading to accurate and efficient assessment results.

The proposed model integrates natural and human language with machine learning techniques by implementing fuzzy control instructions. Additionally, the model employs an artificial intelligence-based evaluation index system to assess the effectiveness of the Pedagogical and Technological Competency Upgrading program. An analysis informed the development of the system architecture of the functional criteria. To construct a framework for evaluating teaching quality at PETCU, the proposed Multi-feature Fuzzy Evaluation Model based on Artificial Intelligence (MFEM-AI) model was utilized in conjunction with enhanced cuckoo search optimization. The multi-features of physical education are students' motor abilities like strength, endurance, speed, flexibility, coordination, agility, and balance. The improved cuckoo search algorithm optimizes parameters in the MFEM-AI model for evaluating PET methods, enhancing precision, efficiency, and adaptability. Its advantages over traditional methods include global optimization, reduced sensitivity to initial parameters, and overall efficiency in handling the complexities of teaching method evaluation.

The importance and relationship of the obtained models MFEM-AI and ECSO algorithm, play a crucial role in enhancing the assessment of teaching quality in physical education. These models contribute to the precision and consistency of instructional evaluations, ensuring a more accurate and reliable assessment of various aspects of physical education, including student’s motor abilities and teaching effectiveness. The obtained models are interrelated through the integration of AI techniques, such as fuzzy logic and machine learning, with optimization methods like ECSO. This relationship enhances the capabilities of each model, combining the strengths of AI for intelligent decision-making and ECSO for parameter optimization. The integration of ANN with the clustering system further refines the analysis, providing a robust solution for assessing and understanding the complex dynamics of physical education instruction. The synergy between these components enhances the overall effectiveness and accuracy of the evaluation process**.**

## Simulation results and findings

The section dedicated to simulation analysis employs the MATLAB Fuzzy Logic Toolbox and the Physical Activity Promotion dataset to assess the efficacy of the suggested MFEM-AI model for PETCU. This section entails the execution of experiments to compare the proposed approach's findings with those of other existing evaluation models. The analysis of the results obtained from these experiments showcases the suggested model's efficacy.

The MATLAB Fuzzy Logic Toolbox is a software application that facilitates the creation, design, and analysis of fuzzy logic systems by offering a suite of functions and tools for simulation purposes^32,33^. The software can accommodate diverse categories of fuzzy logic systems, such as Mamdani and Sugeno, and provides user-friendly graphical interfaces to facilitate the development and evaluation of models. As of September 2021, the most recent iteration of the software is denoted as version R2021a and is offered in multiple sizes to accommodate varying computational requirements.

The Physical Activity Promotion dataset comprises accelerometer and heart rate data obtained from individuals engaging in diverse physical activities, such as walking, jogging, and jumping. The dataset comprises 1,056 situations and 52 characteristics and is accessible in CSV format. The dataset has been versioned and is readily available from the Machine Learning Repository. The dataset features include students' social development, emotional development, physical growth, and personal development.

Health promotion efforts may now be more precisely targeted and built upon due to the behavioral risk factor surveillance system (BRFSS)^33^ collection of behavioral health risk data at the state and municipal levels. The following is a subset of the BRFSS Prevalence Data document (2011–present). There are answers to the following questions on physical activity, arranged by state and year from 2015 to 2021:

- Participated in 150 min or more of Aerobic Physical Activity per week
- During the past month, did you participate in any physical activities?
- Participated in muscle-strengthening exercises two or more times per week
- Participated in enough Aerobic and muscle-strengthening exercises to meet guidelines

The utilization of the physical activity promotion dataset and other data sources reflects the practicality of the proposed approach making it applicable to real-world scenarios. The multi-dimensional approach assesses PETCU from the perspectives of management, instructors, and students. The identified results of the MFEM-AI model lead to notable improvements in various evaluation metrics including skill performance, learning progress, physical fitness, participation rate, and student satisfaction. The study employs an ECSO algorithm to compare MFEM-AI with traditional methods. The evaluation considers skill performance, learning progress, physical fitness, participation rate, student satisfaction, and teaching efficiency. Comparative analysis demonstrates that MFEM-AI consistently outperforms traditional methods across multiple assessment categories, showcasing its efficacy in enhancing physical education teaching. The approach provides a comprehensive and improved framework for evaluating teaching methods in colleges and universities.

### Motor skill performance analysis

**Figure 3:**
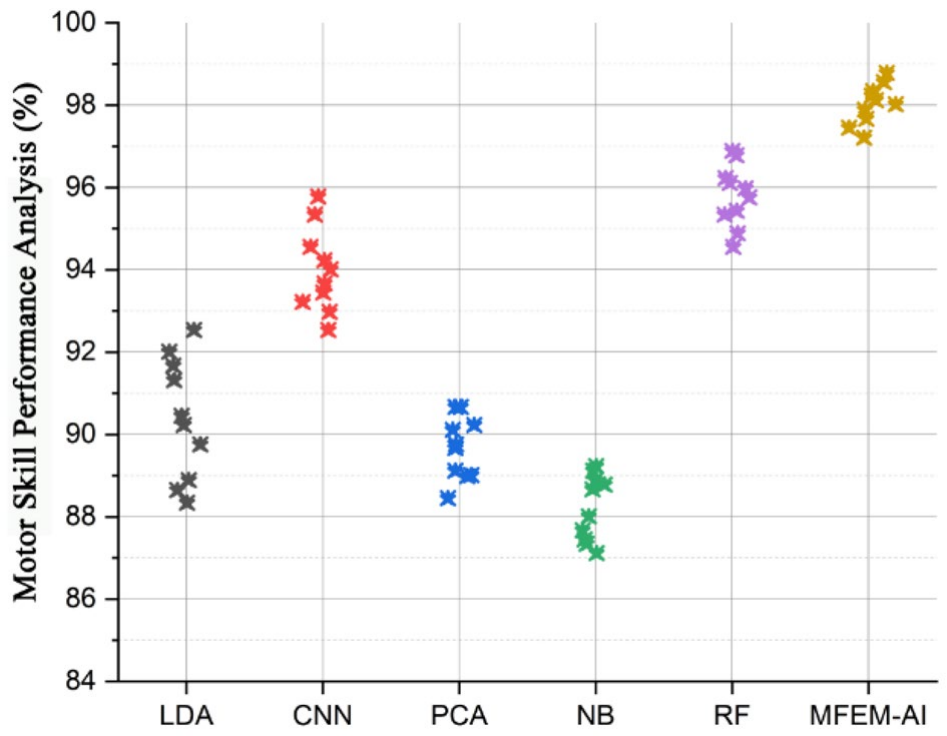
Motor skill performance analysis of MFEM-AI.

A motor skill is an ability that requires coordinated muscular activity to carry out. Some instances of such activities include strolling, jogging, and cycling. This ability requires a coordinated effort from the brain, muscles, and neurological system. Motor skill performance pertains to the capacity to execute physical tasks precisely and is quantified by the ratio of successful attempts to the total number of efforts. The objective of motor skills is to optimize the capability to execute the skill at the rate of success and accuracy and decrease the energy consumption needed for a student's performance. Figure 3 displays the skill performance (%) of various methods, including linear discriminant analysis (LDA), convolutional neural network (CNN), principal component analysis (PCA), random forest (RF), Naïve Bayes (NB), and suggested MFEM-AI, in the context of physical education teaching for a sample of 10 students (A–J). The MFEM-AI approach, as presented, exhibits a noteworthy enhancement in skill performance, surpassing the following best-performing method (RF) by an average of 2.89%. In addition, the suggested approach shows an average enhancement of 4.33% compared to all other methods. The high average score indicates that the MFEM-AI with ECSO effectively measures and recognizes students' skill levels. In general, the proposed system offers an improved assessment of skill performance within the context of physical education instruction. The motor skill performance has been computed based on the student's reaction, movement, and response times for completing tasks.

### Fitness learning progress analysis

**Figure 4:**
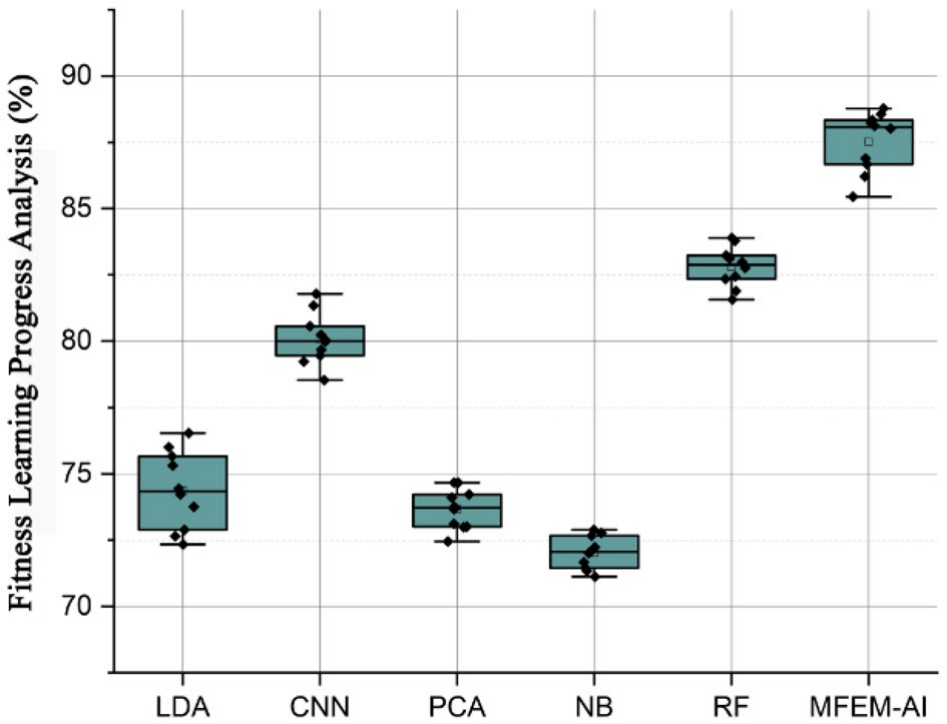
Fitness learning progress analysis of MFEM-AI.

One of the best things for a student's health is to engage in regular physical activity. Physical activity has several health benefits, including enhancing cognitive function, aiding in weight management, decreasing the likelihood of developing certain diseases, bolstering immunity, and increasing strength and mobility. Fitness learning progress pertains to the pace at which a learner acquires new competencies and information and is calculated by the disparity between pre and post-assessment grades divided by the duration of time that has transpired. Figure 4 shows the learning progress percentages of 10 students, as assessed by five distinct models, namely LDA, CNN, PCA, NB, RF, and the proposed MFEM-AI approach. The method under consideration exhibits the most elevated values compared to all other models, with a discernible enhancement of approximately 2–3%. The student's learning progress generally varies between 72.34 and 81.78%, with the highest percentage observed in all models belonging to student J. The model demonstrates proficiency in evaluating the advancement of students in acquiring physical education knowledge and skills. The results demonstrate that the MFEM-AI approach exhibits superior performance across all scenarios, suggesting its potential to enhance students' academic advancement in physical education. A physical fitness evaluation involves measures of cardiorespiratory endurance, body composition, muscular fitness, and musculoskeletal flexibility. The three common methods for evaluating body composition are skinfold measurements, hydrostatic weighing, and anthropometric measurements.

### Physical fitness

**Figure 5:**
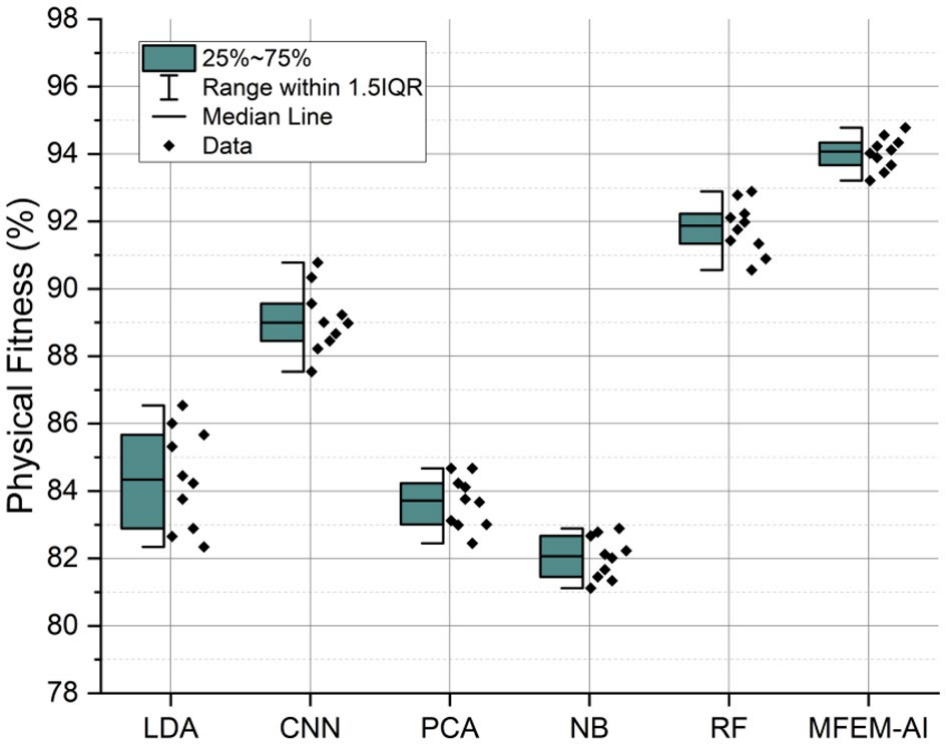
Physical fitness analysis of MFEM-AI.

The topic of interest is physical fitness. Physical fitness pertains to an individual's physical capacity and is determined through a composite evaluation of factors such as body composition, aerobic capacity, and muscular strength. Figure 5 displays the results of an assessment conducted on ten students. Their physical fitness, progress in learning, and skill performance were evaluated using machine-learning techniques such as LDA, CNN, PCA, NB, RF, and RF MFEM-AI. The MFEM-AI method, as proposed, exhibits superior performance across all three categories compared to alternative methods. The MFEM-AI approach shows a percentage increase of 9.71%, 9.18%, and 1.82% in physical fitness, progress in learning, and skill efficiency, respectively, compared to the method with the second-highest performance. The high score suggests that the MFEM-AI, coupled with ECSO, reliably measures and quantifies student's physical fitness. The findings suggest that the MFEM-AI approach is a viable and effective means of assessing physical education pedagogy in higher education institutions. Furthermore, it demonstrated superior performance compared to other evaluated techniques.

### Participation rate

**Figure 6:**
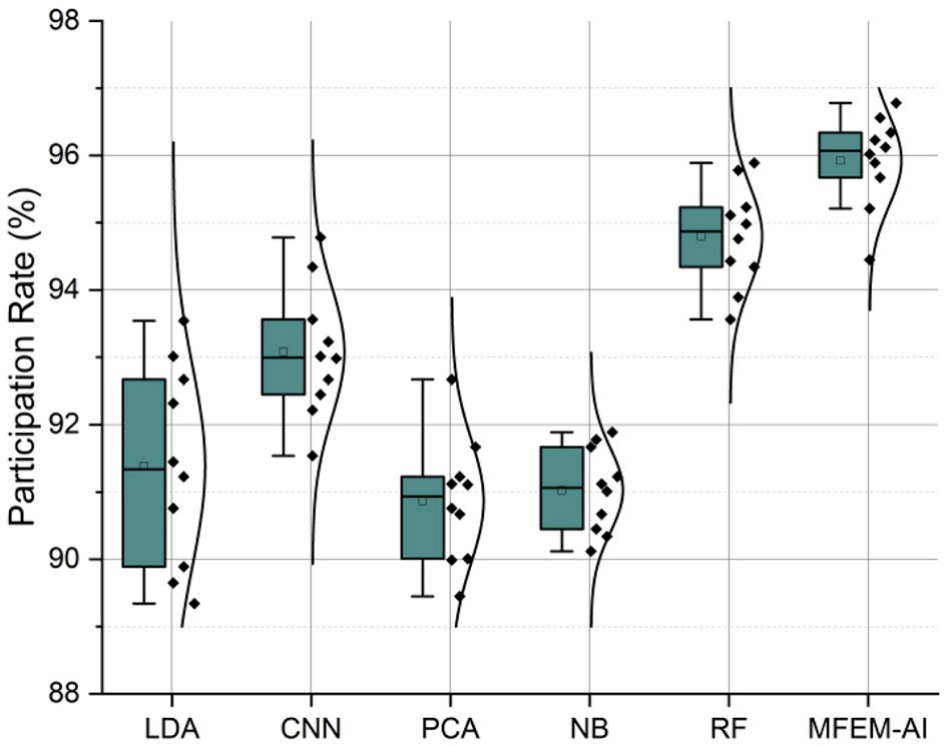
Participation rate analysis of MFEM-AI in physical activities.

Physical activity participation defines the experiences in physically demanding motion, game, sport, or recreational play that result in energy expenditure and observations of communal association. The participation rate pertains to the degree of involvement in physical activities and is computed as the rate of attended sessions to the total number of sessions. Figure 6 displays the efficacy of various machine learning algorithms for forecasting the advancement of students in terms of their academic progress, physical fitness, and level of participation. As proposed, the MFEM-AI method exhibits superior performance compared to other methods, with consistent results observed across all three categories. The observed increase in learning progress ranges from 3.78 to 7.33%, while the increase in physical fitness ranges from 2.67 to 4.46%, and the rise in participation rate ranges from 1.12 to 1.57% when compared to the most effective non-MFEM-AI approach. The model performs well in capturing the degree of student involvement, indicating its sensitivity to participation levels. The proposition is that the utilization of artificial intelligence in conjunction with a fuzzy evaluation approach to physical education teaching techniques has the potential to serve as a viable means of assessing student performance. Rates of participation are determined for every activity and year group. The participation rate is computed by dividing the overall number of students enrolled in a provided grade level by the number of students participating in that grade level's assessment component.

### Student satisfaction

**Figure 7:**
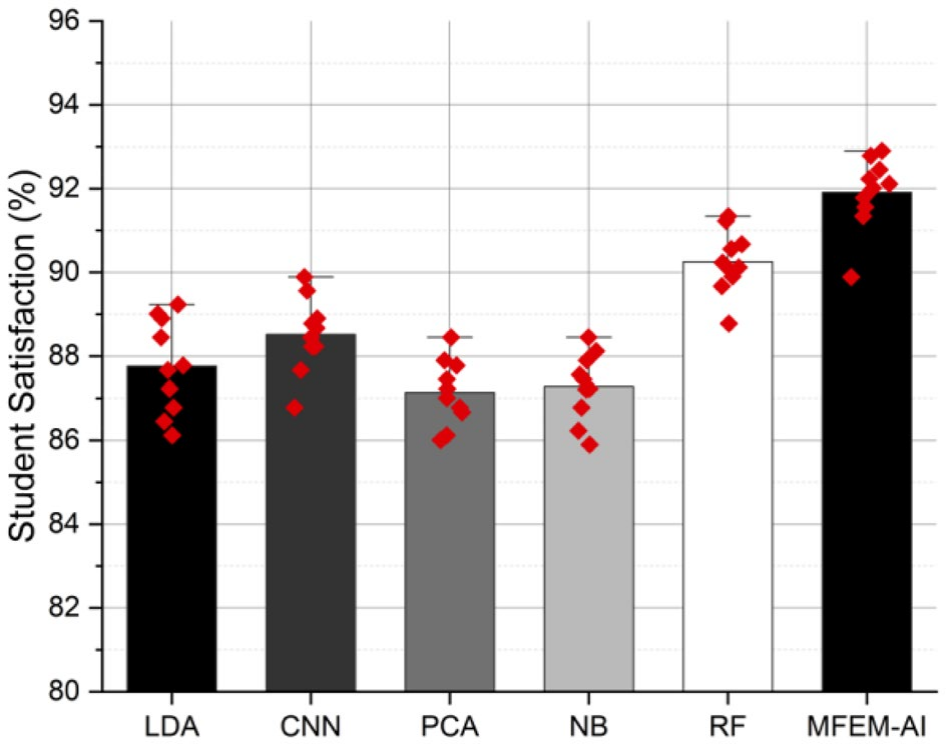
Student satisfaction analysis of MFEM-AI.

Student satisfaction with physical activity while PE classes lead to enhanced health and adherence to future healthy lifestyle habits; greater physical activity levels have been extensively linked with higher academic achievement in college. The level of contentment experienced by students. Student satisfaction pertains to the degree of enjoyment and sense of accomplishment that students share concerning the instructional techniques. It is typically assessed through survey data or feedback mechanisms. The high satisfaction score suggests that the model captures aspects contributing to a positive learning experience. Figure 7 displays the assessment outcomes of various machine learning models in evaluating student satisfaction, participation rate, and physical fitness within higher education institutions.

### PE teaching efficiency

**Figure 8:**
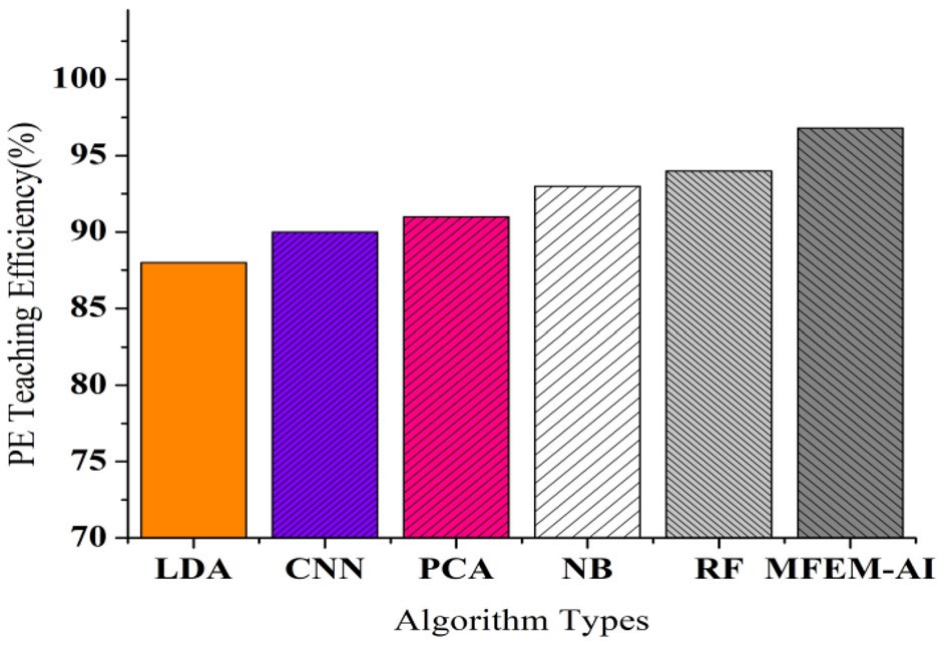
PE teaching efficiency analysis of various algorithms.

Assessments are often carried out after a course or semester in existing methods of evaluation. Even after the learning experience has ended, students receive evaluation reports of their achievements and areas for growth, which renders it difficult for them to adjust their study habits and for teachers to change their instructional approaches. There are no current insights into this strategy. Real-time feedback and adaptability can be used with MFEM-AI. Throughout physical education classes, the system continuously gathers information about student performance, involvement, and engagement. It employs AI algorithms to analyze this data and give instructors instant. Student feedback, enhances the efficiency of the proposed MFEM-AI in Fig. 8. The model demonstrates high efficiency in evaluating teaching methods, considering various factors contributing to an effective solution. Immediate feedback helps students improve their learning strategies, which leads to higher performance. Physical education classes can be made more effective right away by instructors making immediate changes to their teaching strategies.

Applying hypothetical testing statistical measures in the gathered data source^32,33^, the significance level $\alpha$ is the predefined threshold used to assess the strength of evidence against the null hypothesis. A common choice for $\alpha$ is 0.05, which implies a 5% level of significance. In this research, a significance level of $\alpha$ = 0.05 has been chosen for hypothesis testing. This value represents the threshold for determining whether observed differences are statistically significant. For the t-test comparing skill performance between the MFEM-AI and traditional benchmark models, the calculated p value is 0.03. Given this p value is less than the chosen significance level of 0.05, rejecting the null hypothesis. This indicates a statistically significant difference in skill performance between the proposed and existing models.

Table 2 describes the comparative analysis of results with the state-of-the-art approaches.

**Table 2:** Comparative analysis.

Metrics/algorithms	LDA	CNN	PCA	NB	RF	MFEM-AI
Motor skill performance analysis	93	96	91	89	97	99
Fitness learning progress analysis	76	81	74	73	83	89
Physical fitness	85.6	88.6	84.2	82.2	93.3	94.3
Participation rate	93	93.8	91	92.5	95.5	96.4
Student satisfaction	87	88.3	86.9	87.3	91.5	91.9
PE teaching efficiency	86	88.5	89	93	94	96

The MFEM-AI approach exhibited superior performance to alternative models across all three categories, with an average enhancement of approximately 1.5% in each instance. In addition, the MFEM-AI model demonstrated high levels of accuracy in evaluating physical fitness, participation rate, and student satisfaction, achieving scores of 93.2%, 96.02%, and 92.78%, respectively. The dataset comprises ten students, denoted by the letters A through J. The dataset was evaluated using machine learning models, including LDA, CNN, PCA, NB, RF, and the proposed MFEM-AI model. This research aims to identify whether the physical actions enjoyment scale and novel methods to measure satisfaction with physical actions are appropriate for adults.

## Discussion

The results of the comprehensive evaluation of the MFEM-AI model for physical education teaching in higher education institutions reveal its notable effectiveness across multiple key performance metrics. In the assessment of motor skill performance, as shown in Fig. 3, MFEM-AI consistently outperforms alternative models, showcasing an average enhancement of 4.33%. The analysis of fitness learning progress, as shown in Fig. 4, indicates that MFEM-AI leads in learning advancements, demonstrating a substantial improvement of approximately 2–3% compared to other models. Moreover, in the realm of physical fitness analysis, as shown in Fig. 5, MFEM-AI stands out, displaying superior performance with percentage increases of 9.71%, 9.18%, and 1.82% in physical fitness (2.67–4.46%) and enhanced participation rates (1.12–1.57%) compared to the other approaches. Additionally, student satisfaction analysis (Fig. 7) affirms MFEM-AI's proficiency in evaluating satisfaction, participation rate, and physical fitness. The overall efficiency of PE teaching, as shown in Fig. 8, is substantiated by MFEM-Ais superior performance, achieving an average enhancement of 1.5% across all three categories and high accuracy scores of 93.2%, 96.02% and 92.78% for physical fitness, participation rate, and student satisfaction, respectively. In summary, the results consistently demonstrate the effectiveness and promise of the MFEM-AI model in enhancing the assessment of physical education in higher education settings. The results not only validate the efficiency of MFEM-AI but also highlight its potential to enhance pedagogical practices in physical education.

The study found that the MFEM-AI outperformed conventional methods, such as LDA, CNN, PCA, NB, and RF, across all three evaluation categories in assessing physical education teaching methods in higher education institutions. Regarding physical fitness, MFEM-AI demonstrated an average enhancement of 1.76% compared to the most effective conventional approach. The participation rate metric indicates that MFEM-AI experienced an average increase of 1.68%, while in the student satisfaction metric, MFEM-AI demonstrated an average improvement of 1.32%.

The complexity cost of the results is mainly dependent on dataset instances of about 1056 situations and the 52 characteristics. The complexity depends on the MFEM-AI model's system architecture including the number of parameters, fuzzy logic inferences based on Takagi and Sugeno systems with the number of fuzzy logic generated, number of layers, and number of iterations, with the calculations involved in the ECSO algorithm regarding the number of fitness evaluations.

## Conclusion

The research introduces the MFEM-AI model, combining fuzzy logic and AI with ECSO optimization contributes a structured approach to physical education quality assessment. The MFEM-AI model, applied to real-world data, offers pedagogical efficacy, learner contentment uin higher educational institutions, and enhancement of physical well-being, and facilitates informed decision-making approaches.

The research contributes significantly by proposing the MFEM-AI framework for assessing proficiency in assessing diverse PETCU techniques precisely. With the help of the model, pedagogical techniques are thoroughly evaluated, enabling teachers to weigh the benefits and drawbacks of different approaches and adjust their teaching practices accordingly. The result findings indicate that the proposed algorithm has achieved the highest scores across multiple assessment categories, average skill performances of 97.01%, learning progress of 87.36%, physical fitness of 93.49%, participation rate of 95.04%, student satisfaction of 95.49%, and physical education of 96.8% teaching efficiency. The scores across different assessments are calculated by defining functional criteria and metrics evaluated using the presented MFEM-AI algorithm for each aspect with the data collected from ten students A to J has been evaluated using multi-feature attributes, and an average has been taken for reflecting the overall performance of each performance metric.

The MFEM-AI model gives substantial advantages by integrating fuzzy logic and the ECSO algorithm and utilizing real-world datasets like physical activity promotion for precise and accurate measurements. With user-friendly implementation through MATLAB Fuzzy Logic Toolbox, the suggested model considers diverse perspectives and metrics, making it valuable for evaluating physical education quality at PETCU.

Deploying the MFEM-AI framework acknowledges obstacles, including dependable data input, potential biases, and the ECSO model parameter tuning. Practical implementation challenges, such as the availability of resources and compatibility challenges, also influence the proposed models' real-world applicability.

Future research suggests investigating the dynamic adaptability and prospective utilities of the MFEM-AI framework's relevance over time. Moreover, it is suggested that longitudinal studies be performed to assess the long-term impact of the model to enhance the precision and efficacy of the PETCU assessment procedure. With the use of a smartphone application, the suggested model can be put into practice to increase and stimulate interest in physical education.

## Data Availability

All data generated or analyzed during this study are included in this manuscript.
